# Trends in Cannabis Polysubstance Use During Early Pregnancy Among Patients in a Large Health Care System in Northern California

**DOI:** 10.1001/jamanetworkopen.2022.15418

**Published:** 2022-06-06

**Authors:** Kelly C. Young-Wolff, Varada Sarovar, Lue-Yen Tucker, Deborah Ansley, Nancy Goler, Amy Conway, Allison Ettenger, Tara R. Foti, Qiana L. Brown, Ellen T. Kurtzman, Sara R. Adams, Stacey E. Alexeeff

**Affiliations:** 1 Division of Research, Kaiser Permanente Northern California, Oakland; 2 Department of Psychiatry and Behavioral Sciences, University of California, San Francisco; 3 Regional Offices, Kaiser Permanente Northern California, Oakland; 4 School of Social Work, Rutgers, The State University of New Jersey, New Brunswick; 5 School of Nursing, The George Washington University, Washington, DC

## Abstract

**Importance:**

Rates of prenatal cannabis use are increasing alongside perceptions that cannabis is a harmless therapeutic for pregnancy-related ailments, while rates of prenatal use of alcohol and tobacco are decreasing. It is important to examine whether cannabis use during pregnancy is increasing similarly among patients with and patients without co-occurring substance use.

**Objectives:**

To examine trends in cannabis polysubstance use during pregnancy and to test differences in cannabis use over time among pregnant individuals who use only cannabis vs those who use cannabis and other substances.

**Design, Setting, and Participants:**

This cross-sectional time-series study used data from 367 138 pregnancies among 281 590 unique pregnant patients universally screened for prenatal substance use as part of standard care in Kaiser Permanente Northern California from January 1, 2009, to December 31, 2018. Statistical analysis was performed from October 5, 2021, to April 18, 2022.

**Exposures:**

Time (calendar year).

**Main Outcomes and Measures:**

Use of substances during early pregnancy was assessed via universal screening with a self-administered questionnaire (for cannabis, alcohol, stimulants, and nicotine) and/or positive results of a urine toxicology test (for cannabis, alcohol, stimulants, and pharmaceutical opioids), and data were extracted from the electronic health record.

**Results:**

The study sample of 367 138 pregnancies from 281 590 unique pregnant patients (median gestation at time of screening, 8.6 weeks [IQR, 7.3-10.6 weeks]) was 25.9% Asian or Pacific Islander, 6.6% Black, 25.8% Hispanic, 38.0% non-Hispanic White, and 3.6% other race or ethnicity; 1.1% were aged 11 to 17 years, 14.9% were aged 18 to 24 years, 61.9% were aged 25 to 34 years, and 22.1% were aged 35 years or older; and the median neighborhood household income was $70 455 (IQR, $51 563-$92 625). From 2009 to 2018, adjusted rates of use of only cannabis during pregnancy (no other substances) increased substantially from 2.39% (95% CI, 2.20%-2.58%) in 2009 to 6.30% (95% CI, 6.00%-6.60%) in 2018, increasing at an annual relative rate of 1.11 (95% CI, 1.10-1.12). The rate of use of cannabis and 1 other substance also increased (annual relative rate, 1.04 [95% CI, 1.03-1.05]), but not as rapidly (*P* < .001 for difference), while the rate of use of cannabis and 2 or more other substances decreased slightly (annual relative rate, 0.97 [95% CI, 0.96-0.99]). Adjusted rates of prenatal use of cannabis and alcohol (1.04 [95% CI, 1.03-1.06]) and cannabis and stimulants (1.03 [95% CI, 1.01-1.06]) increased over time, while rates of prenatal use of cannabis and nicotine (0.97 [95% CI, 0.96-0.98]) decreased.

**Conclusions and Relevance:**

In this cross-sectional time-series study, rates of prenatal cannabis use during early pregnancy increased significantly more rapidly among patients without co-occurring substance use, which could reflect increased acceptability of cannabis and decreased perceptions of cannabis-related harms. Furthermore, increased rates of use of cannabis with alcohol and stimulants warrant continued monitoring.

## Key Points

**Question:**

Is prenatal cannabis use increasing more rapidly over time among pregnant patients without vs those with co-occurring prenatal substance use?

**Findings:**

In this cross-sectional time-series study using data from 367 138 pregnancies among 281 590 unique pregnant patients screened for prenatal substance use during early pregnancy as part of routine prenatal care in Kaiser Permanente Northern California, rates of prenatal use of only cannabis increased faster than rates of use of cannabis and 1 other substance, while rates of use of cannabis and 2 or more substances decreased.

**Meaning:**

This study suggests that increases in prenatal cannabis use may be associated in part with pregnant individuals who use only cannabis and no other substances, which could reflect growing acceptability of cannabis use and decreasing perceptions of cannabis-related harms.

## Teaser

This cross-sectional study examines trends in cannabis polysubstance use during pregnancy and tests differences in cannabis use over time among pregnant individuals who use only cannabis vs those who use cannabis and other substances.

## Introduction

Cannabis use during pregnancy is a growing public health concern. Prenatal cannabis use is associated with adverse health effects, including poor perinatal outcomes (eg, low birth weight) and neurodevelopmental consequences for children exposed to cannabis in utero.^1,2,3,4,5^ Polysubstance use, a major concern generally in the US,^6^ also has implications for pregnant individuals. During pregnancy, cannabis use increases the risk of other substance use,^7,8,9,10^ which may then compound the adverse health effects of cannabis alone.^11,12,13,14,15^ Tobacco and alcohol are particularly teratogenic,^16,17,18,19,20,21,22,23,24,25^ and their use frequently co-occurs with cannabis use during pregnancy,^7,8^ making it difficult to differentiate health risks specific to prenatal cannabis use vs other substance or polysubstance use.^8^

The American College of Obstetricians and Gynecologists^3^ and the American Academy of Pediatrics^5^ strongly advise pregnant individuals to abstain from cannabis use. However, the prevalence and frequency of prenatal cannabis use have increased in recent years,^26,27,28^ even as rates of prenatal alcohol and nicotine use have decreased.^29,30^ With increased legalization, growing acceptance, and accessibility of cannabis,^31,32^ as well as messages from cannabis retailers that it effectively treats pregnancy-related symptoms (such as morning sickness),^33^ many pregnant individuals using cannabis consider it safe and natural.^34,35^

As prenatal cannabis use rates increase along with perceptions that cannabis is a safe and effective treatment for pregnancy-related symptoms, rates of pregnant women’s co-use of other substances may be decreasing, particularly co-use of tobacco and alcohol. However, we do not know whether prenatal cannabis polysubstance use has changed over time, and research on its changing patterns is needed to better understand prenatal cannabis use and to develop targeted intervention and prevention strategies. Using data from a large, multispecialty health care system in Northern California with universal screening for prenatal substance use, this study examined trends in cannabis polysubstance use from 2009 through 2018, and tested whether prenatal cannabis use was increasing more rapidly among women with vs without co-occurring substance use.

## Methods

### Data Source and Study Population

This cross-sectional time-series study was conducted in a large integrated health care delivery system (ie, Kaiser Permanente Northern California [KPNC]) serving more than 4 million diverse members who are very similar to the fully insured adult population in the Northern California area with regard to race and ethnicity and educational attainment and somewhat less likely to be covered by Medi-Cal or live in a lower-income neighborhood.^36^ All pregnancies screened for prenatal substance use by self-report during standard prenatal care from January 1, 2009, to December 31, 2018, were considered for inclusion. Data were obtained from the electronic health record. The KPNC institutional review board approved this study and waived informed consent because the study uses data only with no participant contact. This study followed the Strengthening the Reporting of Observational Studies in Epidemiology (STROBE) reporting guideline.

### Measures

Pregnant patients in KPNC are universally screened for prenatal substance use, typically during the first prenatal visit (at approximately 8 weeks’ gestation), by both a self-administered questionnaire and a urine toxicology test to which they consent.^37,38,39^ Individuals were classified as having prenatal cannabis, alcohol, or stimulant use if they self-reported any cannabis, alcohol, or stimulant (methamphetamine and cocaine) use since pregnancy and/or had a positive urine toxicology test result for cannabis, alcohol, or stimulants (methamphetamine or amphetamine, cocaine) (eAppendix in the Supplement). Prior work supports combining data from self-report and urine toxicology testing to best capture substance use during pregnancy.^40^ Nicotine use was measured by self-report only (urine toxicology tests for nicotine were not available). Pharmaceutical opioid use was measured by urine toxicology testing only (self-reported data were not available).

We examined any use of each substance (cannabis, alcohol, nicotine, pharmaceutical opioids, and stimulants) and the number of substances co-occurring with cannabis (cannabis only, cannabis and 1 substance, or cannabis and ≥2 substances), and specific type of co-occurring substance use (cannabis and alcohol, cannabis and nicotine, cannabis and pharmaceutical opioids, or cannabis and stimulants).

Our substance use screening (by self-report and urine toxicology) does not distinguish between prescribed vs illicit use of pharmaceutical opioids and amphetamines. We conducted a sensitivity analysis limited to illicit or unauthorized use of these substances by considering use of these substances only among patients who had not filled a prescription for the substance in the 90 days prior to screening. Illicit or unauthorized pharmaceutical opioid use was defined as having a positive result by urine toxicology testing and no prescription for an opioid filled during the 90 days prior to screening. Illicit or unauthorized stimulant use was defined as (1) cocaine and/or methamphetamine use determined by self-report and/or urine toxicology testing, and/or (2) a positive toxicology test result for amphetamines and no prescription for an amphetamine filled during the 90 days prior to the screening date. Sociodemographic variables were extracted from patients’ electronic health record and included age, self-reported race and ethnicity, and median neighborhood household income quartiles based on census data.

### Statistical Analysis

#### Crude Prevalence of Prenatal Cannabis Use and Co-occurring Substance Use

Statistical analysis was performed from October 5, 2021, to April 18, 2022. We calculated the unadjusted prevalence of any prenatal cannabis use, number of substances co-occurring with prenatal cannabis use, and specific type of substance co-occurring with prenatal cannabis use.

#### Linear Trends in Prenatal Cannabis Use and Co-occurring Use of Other Substances

We used Poisson regression with a log-link function to model the annual prevalence and 95% CIs of prenatal cannabis use and polysubstance use. Separate models were run for each outcome: any prenatal cannabis use, number of co-occurring substances, and specific type of co-occurring substance use. Poisson regression is used to model count and rate variables and provides a versatile analytical method for quantifying the time trends. Annual prevalences were adjusted for age, race and ethnicity, and median neighborhood household income using direct standardization to the mean covariate distribution across the study period. Individuals with multiple pregnancies were allowed to contribute to the annual prevalence estimation in multiple years. To model the mean linear trends across the study period, we included a linear term for the calendar year of the first prenatal visit in the Poisson regression model and tested the statistical significance of the trend using a Wald test. To better understand year-by-year changes, we also estimated the relative rate for each study year compared with the previous study year (eg, 2012 vs 2011). Multinomial regression models were conducted to test for the differences in linear trends over time by cannabis polysubstance use (cannabis only, cannabis and 1 substance, cannabis and ≥2 substances).^41^ These multinomial regression models were repeated in sensitivity analyses limited to individuals with illicit or unauthorized use of pharmaceutical opioids or stimulants. Analyses were conducted using SAS, version 9.4 (SAS Institute Inc), and a 2-sided *P* < .05 was considered statistically significant.

## Results

### Study Sample

Of the 418 589 eligible pregnancies, 5033 (1.2%) with an incomplete prenatal substance use screening questionnaire and an additional 46 418 (11.1%) without a urine toxicology test were excluded. The final study sample included 367 138 pregnancies from 281 590 unique pregnant patients screened for prenatal substance use (median gestation at time of screening, 8.6 weeks [IQR, 7.3-10.6 weeks]); 72 012 patients (25.6%) had more than 1 pregnancy during the study period.

The sample of pregnancies was 25.9% Asian or Pacific Islander, 6.6% Black, 25.8% Hispanic, 38.0% non-Hispanic White, and 3.6% other race or ethnicity (American Indian or Alaska Native, those with multiple races, and those with unknown or missing race or ethnicity); 1.1% were aged 11 to 17 years, 14.9% were aged 18 to 24 years, 61.9% were aged 25 to 34 years, and 22.1% were aged 35 years or older; and the median neighborhood household income was $70 455 (IQR, $51 563-$92 625). Differences were minimal between pregnancies excluded vs included in age (mean [SD], 30.4 [5.8] vs 30.2 [5.6] years; *P* < .001) or median neighborhood household income (mean [SD], $73 539 [$31 115] vs $74 299 [$30 712]; *P* < .001); however, pregnancies in Asian or Pacific Islander (11.2%), non-Hispanic White (12.2%), and Hispanic patients (12.6%) were slightly less likely to be excluded than pregnancies in Black patients (14.7%) or those with other or unknown race or ethnicity (14.1%) (*P* < .001).

### Crude Prevalence of Prenatal Cannabis Use and Polysubstance Use

Across 10 years, 6.1% of patients screened positive for any prenatal cannabis use (eTable 1 in the Supplement); 3.4% indicated no cannabis use by self-report but had positive urine toxicology test results, 1.0% indicated cannabis use by self-report but had negative urine toxicology test results, and 1.7% indicated cannabis use by self-report and had positive urine toxicology test results. More than two-thirds of patients who used cannabis (69.6%) used only cannabis, while 22.4% used cannabis and 1 other substance, and 8.0% used cannabis and 2 or more substances. In the overall sample of all pregnant patients, 4.2% used only cannabis, 1.4% used cannabis and 1 other substance, and 0.5% used cannabis and 2 or more substances; 1.2% used cannabis and alcohol, 0.9% used cannabis and nicotine, 0.1% used cannabis and pharmaceutical opioids, and 0.2% used cannabis and stimulants. Prenatal cannabis use and polysubstance use were more common among patients who were younger, Black, and from a lower-income neighborhood (eTable 1 in the Supplement).

Table 1 shows the unadjusted prevalence of any cannabis use, number of co-occurring substances, and specific type of substance co-use across study years. The crude prevalence of any cannabis use increased from 4.52% in 2009 to 8.01% in 2018. This increase appeared to be associated predominantly with an increase in the crude prevalence of only cannabis use, from 2.62% in 2009 to 5.77% in 2018. No trends across cannabis polysubstance use were observed when looking at the crude prevalences.

**Table 1.** Unadjusted Prevalence of Prenatal Cannabis Use During Early Pregnancy in Kaiser Permanente Northern California, by Number of Co-occurring Substances, 2009-2018 (N = 367 138)^a^

Substance use	Unadjusted prevalence of cannabis and co-occurring substance use during pregnancy, %									
	2009	2010	2011	2012	2013	2014	2015	2016	2017	2018
Any cannabis use	4.52	4.93	5.36	5.33	5.41	5.98	6.23	6.54	7.25	8.01
No. of co-occurring substances										
Cannabis only	2.62	3.12	3.42	3.46	3.64	4.24	4.55	4.91	5.47	5.77
Cannabis and 1 other substance	1.29	1.24	1.34	1.32	1.31	1.30	1.27	1.23	1.38	1.78
Cannabis and ≥2 other substances	0.61	0.57	0.59	0.55	0.46	0.44	0.41	0.39	0.40	0.46
Type of co-occurring substance use										
Cannabis only	2.62	3.12	3.42	3.46	3.64	4.24	4.55	4.91	5.47	5.77
Cannabis and alcohol	1.19	1.15	1.19	1.19	1.14	1.12	1.07	1.04	1.28	1.75
Cannabis and nicotine	1.16	1.07	1.12	1.04	0.91	0.88	0.81	0.80	0.71	0.74
Cannabis and pharmaceutical opioids	0.08	0.06	0.07	0.08	0.09	0.09	0.10	0.07	0.07	0.06
Cannabis and stimulants	0.18	0.16	0.21	0.17	0.15	0.18	0.18	0.17	0.18	0.20

^a^ When presenting the crude prevalence for type of co-occurring substance use, with the exception of cannabis only, categories are not mutually exclusive, and patients can be included in more than 1 group (eg, cannabis and alcohol, cannabis and nicotine). Therefore, the categories within a year do not add up to the total prevalence of cannabis use in that year.

### Adjusted Linear Trends in Cannabis Use and Polysubstance Use During Pregnancy

**Figure 1.**
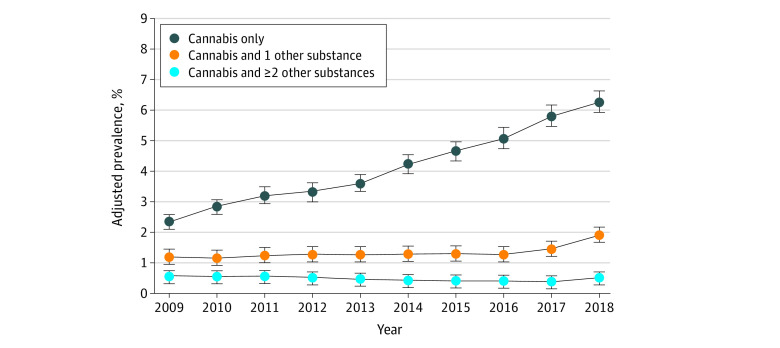
Adjusted Prevalence of Cannabis Use During Early Pregnancy in Kaiser Permanente Northern California, by Count of Polysubstance Use, 2009-2018 (N = 367 138) Error bars indicate 95% CIs.

From 2009 to 2018, the adjusted prevalence of any prenatal cannabis use increased from 4.14% (95% CI, 3.85%-4.43%) to 8.73% (95% CI, 8.33%-9.12%), at a mean annual relative rate of 1.08 (95% CI, 1.08-1.09) (eTable 2 in the Supplement). Use of only cannabis during pregnancy (no other substances) increased substantially from 2.39% (95% CI, 2.20%-2.58%) in 2009 to 6.30% (95% CI, 6.00%-6.60%) in 2018, increasing at a mean annual relative rate of 1.11 (95% CI, 1.10-1.12) (Table 2; Figure 1). Use of cannabis and 1 other substance during pregnancy increased moderately from 1.19% (95% CI, 1.07%-1.32%) in 2009 to 1.92% (95% CI, 1.77%-2.07%) in 2018 at a mean annual relative rate of 1.04 (95% CI, 1.03-1.05). Comparing the annual rates of increase, use of only cannabis during pregnancy increased faster than use of cannabis and 1 other substance during pregnancy (*P* < .001). Annual rates of use of cannabis and 1 other substance during pregnancy increased more sharply in recent years, with a relative rate of increase of 1.15 (95% CI, 1.00-1.31) for 2017 vs 2016 and a relative rate of increase of 1.31 (95% CI, 1.16-1.48) for 2018 vs 2017. Use of cannabis and 2 or more other substances during pregnancy decreased slightly from 0.56% (95% CI, 0.48%-0.64%) in 2009 to 0.50% (95% CI, 0.43%-0.57%) in 2018 at a mean annual relative rate of 0.97 (95% CI, 0.96-0.99).

**Table 2.** Adjusted Prevalence of Cannabis Use During Early Pregnancy in Kaiser Permanente Northern California for Each Year (2009-2018) and Annual Relative Rate of Change, by Polysubstance Use (N = 367 138)

Substance use	Adjusted prevalence of cannabis and co-occurring substance use during pregnancy, % (95% CI)^a^											Linear trend estimation	
	2009	2010	2011	2012	2013	2014	2015	2016	2017	2018	Annual relative rate of change estimate (95% CI)		*P* value
**No. of co-occurring substances**													
Cannabis only	2.39 (2.20-2.58)	2.87 (2.65-3.08)	3.23 (3.00-3.46)	3.34 (3.11-3.57)	3.63 (3.38-3.87)	4.24 (3.99-4.50)	4.70 (4.43-4.97)	5.11 (4.84-5.39)	5.87 (5.57-6.16)	6.30 (6.00-6.60)	1.11 (1.10-1.12)		<.001
Annual relative rate	NA	1.20 (1.07-1.34)	1.13 (1.02-1.25)	1.03 (0.94-1.14)	1.08 (0.99-1.19)	1.17 (1.07-1.28)	1.11 (1.02-1.20)	1.09 (1.01-1.18)	1.15 (1.07-1.23)	1.07 (1.00-1.15)			
Cannabis and 1 other substance	1.19 (1.07-1.32)	1.16 (1.03-1.28)	1.27 (1.14-1.40)	1.28 (1.15-1.42)	1.30 (1.16-1.43)	1.30 (1.17-1.43)	1.31 (1.18-1.44)	1.28 (1.16-1.41)	1.47 (1.33-1.60)	1.92 (1.77-2.07)	1.04 (1.03-1.05)		<.001
Annual relative rate	NA	0.97 (0.83-1.13)	1.10 (0.95-1.28)	1.01 (0.87-1.17)	1.01 (0.87-1.17)	1.00 (0.87-1.16)	1.01 (0.87-1.16)	0.98 (0.85-1.13)	1.15 (1.00-1.31)	1.31 (1.16-1.48)			
Cannabis and ≥2 other substances	0.56 (0.48-0.64)	0.53 (0.45-0.60)	0.56 (0.48-0.64)	0.53 (0.46-0.61)	0.45 (0.38-0.53)	0.44 (0.37-0.51)	0.43 (0.36-0.49)	0.41 (0.35-0.48)	0.43 (0.36-0.50)	0.50 (0.43-0.57)	0.97 (0.96-0.99)		<.001
Annual relative rate	NA	0.94 (0.77-1.15)	1.06 (0.86-1.29)	0.95 (0.78-1.17)	0.85 (0.69-1.06)	0.96 (0.77-1.20)	0.97 (0.78-1.22)	0.97 (0.78-1.21)	1.04 (0.84-1.30)	1.16 (0.94-1.43)			
**Type of co-occurring substance use**													
Cannabis only	2.39 (2.20-2.58)	2.87 (2.65-3.08)	3.23 (3.00-3.46)	3.34 (3.11-3.57)	3.63 (3.38-3.87)	4.24 (3.99-4.50)	4.70 (4.43-4.97)	5.11 (4.84-5.39)	5.87 (5.57-6.16)	6.30 (6.00-6.60)	1.11 (1.10-1.12)		<.001
Annual relative rate	NA	1.20 (1.07-1.34)	1.13 (1.02-1.25)	1.03 (0.94-1.14)	1.08 (0.99-1.19)	1.17 (1.07-1.28)	1.11 (1.02-1.20)	1.09 (1.01-1.18)	1.15 (1.07-1.23)	1.07 (1.00-1.15)			
Cannabis and alcohol	1.11 (0.99-1.23)	1.08 (0.96-1.21)	1.14 (1.01-1.27)	1.16 (1.03-1.28)	1.13 (1.01-1.26)	1.12 (0.99-1.24)	1.10 (0.98-1.22)	1.07 (0.96-1.19)	1.35 (1.22-1.48)	1.87 (1.73-2.02)	1.04 (1.03-1.06)		<.001
Annual relative rate	NA	0.97 (0.83-1.14)	1.05 (0.90-1.23)	1.02 (0.87-1.19)	0.98 (0.84-1.14)	0.99 (0.84-1.15)	0.99 (0.85-1.15)	0.97 (0.84-1.13)	1.26 (1.09-1.45)	1.39 (1.23-1.57)			
Cannabis and nicotine	1.05 (0.93-1.16)	0.97 (0.86-1.09)	1.05 (0.93-1.17)	0.99 (0.88-1.11)	0.90 (0.79-1.01)	0.88 (0.77-0.98)	0.85 (0.74-0.95)	0.84 (0.74-0.94)	0.78 (0.68-0.88)	0.83 (0.73-0.92)	0.97 (0.96-0.98)		<.001
Annual relative rate	NA	0.93 (0.79-1.09)	1.07 (0.91-1.26)	0.95 (0.81-1.11)	0.91 (0.77-1.07)	0.98 (0.82-1.16)	0.96 (0.81-1.15)	0.99 (0.84-1.18)	0.93 (0.78-1.10)	1.06 (0.89-1.26)			
Cannabis and pharmaceutical opioids	0.08 (0.06-0.10)	0.06 (0.04-0.08)	0.07 (0.05-0.09)	0.08 (0.06-0.10)	0.09 (0.07-0.11)	0.09 (0.07-0.11)	0.10 (0.08-0.12)	0.07 (0.06-0.09)	0.07 (0.05-0.08)	0.07 (0.05-0.08)	1.00 (0.97-1.03)		.95
Annual relative rate	NA	0.81 (0.56-1.19)	1.15 (0.78-1.70)	1.08 (0.75-1.56)	1.16 (0.82-1.63)	0.95 (0.68-1.32)	1.20 (0.88-1.64)	0.73 (0.53-1.00)	0.91 (0.64-1.28)	0.97 (0.68-1.38)			
Cannabis and stimulants	0.16 (0.12-0.20)	0.15 (0.11-0.18)	0.20 (0.16-0.24)	0.17 (0.13-0.21)	0.15 (0.11-0.19)	0.18 (0.14-0.21)	0.18 (0.14-0.22)	0.18 (0.14-0.22)	0.20 (0.16-0.24)	0.23 (0.18-0.27)	1.03 (1.01-1.06)		.01
Annual relative rate	NA	0.90 (0.65-1.26)	1.37 (0.99-1.88)	0.84 (0.62-1.14)	0.90 (0.65-1.26)	1.16 (0.84-1.61)	1.04 (0.77-1.41)	0.98 (0.73-1.32)	1.10 (0.82-1.47)	1.15 (0.87-1.51)			

^a^ Adjusted prevalence estimates and 95% CIs were estimated from Poisson regression models controlling for age group, race and ethnicity, and median neighborhood household income (extracted from the electronic health record).

**Figure 2.**
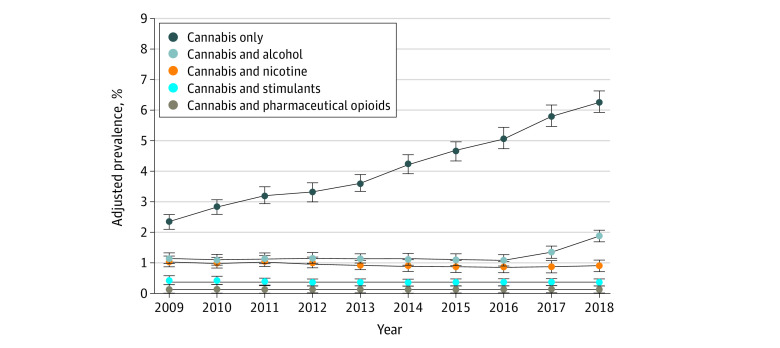
Adjusted Prevalence of Cannabis Use During Early Pregnancy in Kaiser Permanente Northern California, by Type of Polysubstance Use, 2009-2018 (N = 367 138) Error bars indicate 95% CIs.

Regarding specific types of polysubstance use, prenatal co-use of cannabis and alcohol increased from 1.11% (95% CI, 0.99%-1.23%) in 2009 to 1.87% (95% CI, 1.73%-2.02%) in 2018 at a mean annual relative rate of 1.04 (95% CI, 1.03-1.06) (Table 2; Figure 2). This increasing trend appeared to be associated with sharp increases in 2017 and 2018 (relative rates of increase, 1.26 [95% CI, 1.09-1.45] for 2017 vs 2016 and 1.39 [95% CI, 1.23-1.57] for 2018 vs 2017). Prenatal co-use of cannabis and stimulants increased from 0.16% (95% CI, 0.12%-0.20%) in 2009 to 0.23% (95% CI, 0.18%-0.27%) in 2018 at a mean annual relative rate of 1.03 (95% CI, 1.01-1.06). In contrast, prenatal co-use of cannabis and nicotine decreased from 1.05% (95% CI, 0.93%-1.16%) in 2009 to 0.83% (95% CI, 0.73%-0.92%) in 2018 at a mean annual relative rate of 0.97 (95% CI, 0.96-0.98). Prenatal co-use of cannabis and pharmaceutical opioids remained stable, with a prevalence of 0.08% (95% CI, 0.06%-0.10%) in 2009 and 0.07% (95% CI, 0.05%-0.08%) in 2018 and with a mean annual relative rate of 1.00 (95% CI, 0.97-1.03).

### Linear Trends in Other Substance Use During Pregnancy

To better understand the context of trends in cannabis polysubstance use, we separately examined the overall crude prevalence and adjusted trends in other types of prenatal substance use during the study period. Across years, the crude prevalence of prenatal alcohol use (9.78%) was higher than prenatal nicotine use (2.79%), prenatal pharmaceutical opioid use (0.42%), and prenatal stimulant use (0.38%). The prevalence of any prenatal alcohol use, nicotine use, and pharmaceutical opioid use all decreased over time. Alcohol use decreased from 11.73% (95% CI, 11.31%-12.14%) in 2009 to 10.86% (95% CI, 10.51%-11.20%) in 2018 at a mean annual relative rate of 0.98 (95% CI, 0.97-0.98), nicotine use decreased from 4.21% (95% CI, 3.95%-4.48%) in 2009 to 2.06% (95% CI, 1.89%-2.22%) in 2018 at a mean annual relative rate of 0.92 (95% CI, 0.91-0.93), and pharmaceutical opioid use decreased from 0.52% (95% CI, 0.45%-0.58%) in 2009 to 0.22% (95% CI, 0.19%-0.26%) in 2018 at a mean annual relative rate of 0.94 (95% CI, 0.92-0.95) (eTable 2 in the Supplement). In contrast, there was an increase in stimulant use from 0.34% (95% CI, 0.28%-0.40%) in 2009 to 0.48% (95% CI, 0.42%-0.55%) in 2018 at a mean annual relative rate of 1.05 (95% CI, 1.03-1.06).

### Sensitivity Analysis

Of the 1529 pregnancies with positive results for a pharmaceutical opioid via urine toxicology testing, 568 were considered to have illicit or unauthorized pharmaceutical opioid use. Of the 1380 pregnancies with positive results for stimulant use, 1278 were considered to have illicit or unauthorized stimulant use. Using these new definitions, the adjusted linear trends in cannabis polysubstance use (cannabis and 1 substance, cannabis and ≥2 substances) and co-use of cannabis and illicit or unauthorized stimulants changed minimally (eTable 3 in the Supplement). However, in contrast to our primary results, co-use of cannabis and illicit or unauthorized pharmaceutical opioids increased over time, with a prevalence of 0.01% (95% CI, 0.01%-0.02%) in 2009 and 0.04% (95% CI, 0.03%-0.06%) in 2018, and a mean annual relative rate of 1.09 (95% CI, 1.06-1.13).

The prevalence of illicit or unauthorized pharmaceutical opioid use remained stable over time (from 0.14% [95% CI, 0.10%-0.17%] in 2009 to 0.14% [95% CI, 0.11%-0.16%] in 2018), at a mean annual relative rate of 1.02 (95% CI, 1.00-1.05), and the prevalence of illicit or unauthorized stimulant use increased over time (from 0.33% [95% CI, 0.27%-0.39%] in 2009 to 0.41% [95% CI, 0.35%-0.47%] in 2018), at a mean annual relative rate of 1.03 (95% CI, 1.01-1.05).

## Discussion

Our cross-sectional time-series study of trends in cannabis polysubstance use during early pregnancy in a large health care system in Northern California has 4 key findings. First, increases in cannabis use were associated largely with increased cannabis use only, without co-occurring substance use. Second, in contrast to increases in use of only cannabis and use of cannabis and 1 other substance, use of cannabis and 2 or more other substances decreased. Third, trends in prenatal cannabis polysubstance use varied with substance type and whether pharmaceutical opioids were prescribed. Fourth, we saw a reduction in prenatal use of alcohol, nicotine, and pharmaceutical opioids over the 10 years.

Although studies have documented increases in prenatal cannabis use over the past decade, this is the first study, to our knowledge, to show that prenatal cannabis use is increasing significantly more rapidly without co-occurring substance use. Cannabis-only users represented 58% of all patients with prenatal cannabis use in 2009, which increased to 72% of such patients in 2018. Prior studies showed that some pregnant individuals believe that prenatal cannabis is safe and effective to treat pregnancy-related symptoms, such as depression, stress, and morning sickness,^33,42,43,44^ and many individuals who use cannabis during pregnancy perceive slight to no risk in its use.^34,35^ Increases in prenatal cannabis use support the hypothesis that individuals who would otherwise not use any substances during pregnancy may be increasingly turning to cannabis. Alternatively, increases in use of only cannabis during pregnancy could reflect substitution of cannabis for substances viewed as more harmful when used during pregnancy (eg, pharmaceutical opioids or nicotine use, which decreased during the study period). Future research is needed to understand the mechanisms underlying the substantial increase in use of only cannabis without other substances.

Some adverse health effects associated with prenatal cannabis use may be due, in part, to concurrent prenatal use of tobacco and other substances, and prior research has typically lacked power to examine a subgroup of individuals who use only cannabis.^8^ As the use of cannabis without co-occurring substance use becomes increasingly common, it may be easier for researchers to identify the health effects associated specifically with prenatal cannabis use.

We also found a moderate increase in use of cannabis and 1 other substance. Trends in co-use differed depending on substance type, with an increase in co-use of cannabis and alcohol and cannabis and stimulants, and a decrease in co-use of cannabis and nicotine. Although increases in co-use of cannabis and stimulants could reflect, in part, general increases seen in the prevalence of both substances, pregnant patients were less likely to use alcohol, suggesting that increases in co-use of cannabis and alcohol do not simply reflect changes in trends of prenatal alcohol use. The increasing rates of prenatal co-use of cannabis with alcohol and stimulants warrant continued monitoring. In addition, the decreases seen in prenatal use of alcohol, nicotine, and pharmaceutical opioids over the 10 years may partly result from public health campaigns and increasing awareness of the harms associated with prenatal use.^45,46^ However, our sensitivity analysis found an increasing trend of prenatal cannabis and illicit or unauthorized pharmaceutical opioid rates, reflecting increases in co-use of cannabis with pharmaceutical opioids that were not prescribed by a medical professional (eg, via medication diversion).

We found a higher prevalence of cannabis use and cannabis polysubstance use among patients who were younger, Black, or from a lower-income neighborhood, consistent with earlier research demonstrating a higher prevalence of cannabis use among pregnant and nonpregnant patients in these demographic groups.^28,35,47,48^ Future quantitative and qualitative studies are needed to better understand underlying differences in prenatal cannabis use and polysubstance use.

Prior studies found that frequency of prenatal cannabis use is increasing, with daily use increasing most rapidly.^28^ Increases in daily use could reflect increases in acceptability of cannabis or perceptions of safety, decreasing concerns about legal repercussions, or possibly greater addiction due to availability of higher-potency cannabis products. Prior research with KPNC pregnant patients showed increased cannabis use both by self-report and by urine toxicology testing, indicating that increased use is due not only to increased willingness in recent years to disclose use.^27^ Qualitative studies indicate that pregnant individuals are dissatisfied with the quality of information available about health risks of prenatal cannabis use,^34,42^ and report that obstetric health care professionals often do not respond to or counsel patients when they disclose prenatal cannabis use.^49^ Furthermore, individuals who serve customers at cannabis dispensaries (“budtenders”) may spread misinformation by advising that cannabis use during pregnancy is safe for treating pregnancy-related symptoms.^33^ Results from this study highlight the importance of educating health care professionals about the risks of prenatal cannabis use and providing training in how to provide sensitive, patient-centered counseling that helps pregnant patients make informed choices around cannabis use during pregnancy. Public health approaches, such as budtender trainings and more visible package warnings, may be helpful strategies to increase public awareness of the risks associated with prenatal cannabis use and to dispel the misperception that prenatal cannabis use is without harms.

### Limitations

This study has some limitations. Our sample is limited to pregnant patients screened for prenatal substance use during early pregnancy in 1 large integrated health care system in Northern California, and findings may not be generalizable to patients without access to health care, those outside California, or those within KPNC who were not screened for substance use. Although patients screened vs not screened were generally similar, those not screened were slightly more likely to be Black or of other or unknown race and ethnicity. Restricting only to patients who are screened could introduce bias in effect estimates; however, a missing urine toxicology test result is commonly due to system issues such as an insufficient urine sample, and we observed only minor differences in the excluded and included patients, so we expect that our exclusions would introduce minimal bias. Although California’s cannabis legal landscape and use patterns are not representative of the US more broadly, California acts as a bellwether of what is to come for other states, and the current study’s results can help inform future US trends.

Prenatal substance use was assessed at entrance to prenatal care (at approximately 8 weeks’ gestation) and does not reflect continued use. Furthermore, we are unable to distinguish use during early pregnancy that occurred only before vs after patients learned they were pregnant. Although cannabis, cocaine, and methamphetamine use during pregnancy were assessed by self-report and urine toxicology testing, alcohol and nicotine use during pregnancy were limited to patient self-report, and pharmaceutical opioid use and amphetamine use were limited to urine toxicology testing. Owing to variation in the detection window for different substances and limitations of self-report, some patients who used these substances during pregnancy may be misclassified as nonusers. Our study did not measure nonpharmaceutical opioid use (eg, heroin). Furthermore, we were not able to account for frequency of use, quantity of use, or potency of cannabis products. We attempted to differentiate use of stimulants and pharmaceutical opioids that were licit vs unauthorized or illicit using data on past 90-day prescription fills in KPNC; however, some patients may have received prescriptions outside of KPNC. Furthermore, we were unable to assess whether prenatal cannabis use was for medical purposes as recommended by a physician. Additional research is needed to examine patterns of cannabis polysubstance use and substance use disorders throughout pregnancy, and to test whether findings vary depending on whether substance use is self-reported or identified only via results of urine toxicology testing.

## Conclusions

As cannabis use becomes more acceptable and accessible,^31,50^ this cross-sectional time-series study found that its use during early pregnancy is increasing most rapidly among patients who use only cannabis and no other substances. Increasing use of cannabis without other substance use could reflect increasing rates of use among those not at risk for other prenatal substance use or substitution of cannabis for other substances viewed as more harmful when used during pregnancy. Evidence-based public health campaigns to increase education and awareness and more visible package warnings about the potential harms of prenatal cannabis use may enable pregnant individuals to make informed decisions about using cannabis.

## Notes

Supplement. **eAppendix.** Urine Toxicology Testing Description and Positive Cutoffs **eTable 1.** Prevalence of Cannabis Use and Polysubstance Use During Early Pregnancy in KPNC by Sociodemographics, 2009-2018 (N = 367,138) **eTable 2.** Adjusted Prevalence and Annual Relative Rate of Change of Cannabis, Alcohol, Nicotine, Pharmaceutical Opioid, and Stimulant Use During Early Pregnancy in KPNC, 2009-2018 (N = 367,138) **eTable 3.** Sensitivity Analysis of Adjusted Prevalence and Annual Relative Rate of Change of Cannabis Use During Early Pregnancy in KPNC for Each Year (2009-2018), by Polysubstance Use (N = 367,138)

## References

[1] Metz TD, Allshouse AA, Hogue CJ, et al. Maternal marijuana use, adverse pregnancy outcomes, and neonatal morbidity. Am J Obstet Gynecol. 2017;217(4):478.e1-478.e8. doi:10.1016/j.ajog.2017.05.050 28578174 PMC5614818

[2] Sharapova SR, Phillips E, Sirocco K, Kaminski JW, Leeb RT, Rolle I. Effects of prenatal marijuana exposure on neuropsychological outcomes in children aged 1-11 years: a systematic review. Paediatr Perinat Epidemiol. 2018;32(6):512-532. doi:10.1111/ppe.12505 30335203 PMC6261687

[3] Committee on Obstetric Practice. Committee opinion no. 722: marijuana use during pregnancy and lactation. Obstet Gynecol. 2017;130(4):e205-e209. doi:10.1097/AOG.0000000000002354 28937574

[4] National Academies of Sciences, Engineering, and Medicine. The Health Effects of Cannabis and Cannabinoids: The Current State of Evidence and Recommendations for Research. National Academies Press; 2017. 28182367

[5] Ryan SA, Ammerman SD, O’Connor ME; Committee on Substance Use and Prevention; Section on Breastfeeding. Marijuana use during pregnancy and breastfeeding: implications for neonatal and childhood outcomes. Pediatrics. 2018;142(3):e20181889. doi:10.1542/peds.2018-1889 30150209

[6] Jalal H, Buchanich JM, Roberts MS, Balmert LC, Zhang K, Burke DS. Changing dynamics of the drug overdose epidemic in the United States from 1979 through 2016. Science. 2018;361(6408):456-461. doi:10.1126/science.aau1184 30237320 PMC8025225

[7] Coleman-Cowger VH, Schauer GL, Peters EN. Marijuana and tobacco co-use among a nationally representative sample of US pregnant and non-pregnant women: 2005-2014 National Survey on Drug Use and Health findings. Drug Alcohol Depend. 2017;177:130-135. doi:10.1016/j.drugalcdep.2017.03.025 28599211

[8] Goler N, Conway A, Young-Wolff KC. Data are needed on the potential adverse effects of marijuana use in pregnancy. Ann Intern Med. 2018;169(7):492-493. doi:10.7326/M18-1141 30140934 PMC6419734

[9] Jarlenski M, Krans EE. Co-occurring substance use disorders identified among delivery hospitalizations in the United States. J Addict Med. 2021;15(6):504-507. doi:10.1097/ADM.0000000000000792 33273252 PMC8166954

[10] Metz VE, Brown QL, Martins SS, Palamar JJ. Characteristics of drug use among pregnant women in the United States: opioid and non-opioid illegal drug use. Drug Alcohol Depend. 2018;183:261-266. doi:10.1016/j.drugalcdep.2017.11.010 29310077 PMC5803362

[11] Black M, Bhattacharya S, Fairley T, Campbell DM, Shetty A. Outcomes of pregnancy in women using illegal drugs and in women who smoke cigarettes. Acta Obstet Gynecol Scand. 2013;92(1):47-52. doi:10.1111/j.1600-0412.2012.01519.x 22913319

[12] Peters EN, Budney AJ, Carroll KM. Clinical correlates of co-occurring cannabis and tobacco use: a systematic review. Addiction. 2012;107(8):1404-1417. doi:10.1111/j.1360-0443.2012.03843.x 22340422 PMC3377777

[13] Tzilos GK, Reddy MK, Caviness CM, Anderson BJ, Stein MD. Getting higher: co-occurring drug use among marijuana-using emerging adults. J Addict Dis. 2014;33(3):202-209. doi:10.1080/10550887.2014.950024 25115183 PMC4224674

[14] Varner MW, Silver RM, Rowland Hogue CJ, ; Eunice Kennedy Shriver National Institute of Child Health and Human Development Stillbirth Collaborative Research Network. Association between stillbirth and illicit drug use and smoking during pregnancy. Obstet Gynecol. 2014;123(1):113-125. doi:10.1097/AOG.0000000000000052 24463671 PMC3931517

[15] Bailey BA, McCook JG, Hodge A, McGrady L. Infant birth outcomes among substance using women: why quitting smoking during pregnancy is just as important as quitting illicit drug use. Matern Child Health J. 2012;16(2):414-422. doi:10.1007/s10995-011-0776-y 21424740

[16] Dejong K, Olyaei A, Lo JO. Alcohol use in pregnancy. Clin Obstet Gynecol. 2019;62(1):142-155. doi:10.1097/GRF.0000000000000414 30575614 PMC7061927

[17] McQuire C, Daniel R, Hurt L, Kemp A, Paranjothy S. The causal web of foetal alcohol spectrum disorders: a review and causal diagram. Eur Child Adolesc Psychiatry. 2020;29(5):575-594. doi:10.1007/s00787-018-1264-3 30648224 PMC7250957

[18] Meyer-Leu Y, Lemola S, Daeppen JB, Deriaz O, Gerber S. Association of moderate alcohol use and binge drinking during pregnancy with neonatal health. Alcohol Clin Exp Res. 2011;35(9):1669-1677. doi:10.1111/j.1530-0277.2011.01513.x 21554334

[19] Moise IK. Alcohol use, pregnancy and associated risk factors: a pilot cross-sectional study of pregnant women attending prenatal care in an urban city. BMC Pregnancy Childbirth. 2019;19(1):472. doi:10.1186/s12884-019-2652-5 31805891 PMC6896278

[20] O’Leary CM, Nassar N, Kurinczuk JJ, Bower C. The effect of maternal alcohol consumption on fetal growth and preterm birth. BJOG. 2009;116(3):390-400. doi:10.1111/j.1471-0528.2008.02058.x 19187371

[21] Crume T. Tobacco use during pregnancy. Clin Obstet Gynecol. 2019;62(1):128-141. doi:10.1097/GRF.0000000000000413 30668557

[22] American College of Obstetricians and Gynecologists. Tobacco and nicotine cessation during pregnancy: ACOG Committee opinion summary, number 807. Obstet Gynecol. 2020;135(5):1244-1246. doi:10.1097/AOG.0000000000003825 32332411

[23] Gaysina D, Fergusson DM, Leve LD, . Maternal smoking during pregnancy and offspring conduct problems: evidence from 3 independent genetically sensitive research designs. JAMA Psychiatry. 2013;70(9):956-963. doi:10.1001/jamapsychiatry.2013.127 23884431 PMC3828999

[24] Moore BF, Shapiro AL, Wilkening G, . Prenatal exposure to tobacco and offspring neurocognitive development in the Healthy Start Study. J Pediatr. 2020;218:28-34. doi:10.1016/j.jpeds.2019.10.056 31759580 PMC7042047

[25] Oga EA, Mark K, Coleman-Cowger VH. Cigarette smoking status and substance use in pregnancy. Matern Child Health J. 2018;22(10):1477-1483. doi:10.1007/s10995-018-2543-9 29882032 PMC6430977

[26] Brown QL, Sarvet AL, Shmulewitz D, Martins SS, Wall MM, Hasin DS. Trends in marijuana use among pregnant and nonpregnant reproductive-aged women, 2002-2014. JAMA. 2017;317(2):207-209. doi:10.1001/jama.2016.17383 27992619 PMC5595220

[27] Young-Wolff KC, Tucker LY, Alexeeff S, . Trends in self-reported and biochemically tested marijuana use among pregnant females in California from 2009-2016. JAMA. 2017;318(24):2490-2491. doi:10.1001/jama.2017.17225 29279917 PMC5769923

[28] Young-Wolff KC, Sarovar V, Tucker LY, . Self-reported daily, weekly, and monthly cannabis use among women before and during pregnancy. JAMA Netw Open. 2019;2(7):e196471. doi:10.1001/jamanetworkopen.2019.6471 31322686 PMC6646980

[29] Young-Wolff KC, Sarovar V, Alexeeff SE, . Trends and correlates of self-reported alcohol and nicotine use among women before and during pregnancy, 2009-2017. Drug Alcohol Depend. 2020;214:108168. doi:10.1016/j.drugalcdep.2020.108168 32736316 PMC7423641

[30] Agrawal A, Rogers CE, Lessov-Schlaggar CN, Carter EB, Lenze SN, Grucza RA. Alcohol, cigarette, and cannabis use between 2002 and 2016 in pregnant women from a nationally representative sample. JAMA Pediatr. 2019;173(1):95-96. doi:10.1001/jamapediatrics.2018.3096 30398527 PMC6500767

[31] LaBrie JW, Hummer JF, Neighbors C, Pedersen ER. Live interactive group–specific normative feedback reduces misperceptions and drinking in college students: a randomized cluster trial. Psychol Addict Behav. 2008;22(1):141-148. doi:10.1037/0893-164X.22.1.141 18298241 PMC4221269

[32] Jarlenski M, Koma JW, Zank J, Bodnar LM, Bogen DL, Chang JC. Trends in perception of risk of regular marijuana use among US pregnant and nonpregnant reproductive-aged women. Am J Obstet Gynecol. 2017;217(6):705-707. doi:10.1016/j.ajog.2017.08.015 28843740 PMC5971084

[33] Dickson B, Mansfield C, Guiahi M, . Recommendations from cannabis dispensaries about first-trimester cannabis use. Obstet Gynecol. 2018;131(6):1031-1038. doi:10.1097/AOG.0000000000002619 29742676 PMC5970054

[34] Chang JC, Tarr JA, Holland CL, . Beliefs and attitudes regarding prenatal marijuana use: perspectives of pregnant women who report use. Drug Alcohol Depend. 2019;196:14-20. doi:10.1016/j.drugalcdep.2018.11.028 30658220 PMC6756431

[35] Ko JY, Farr SL, Tong VT, Creanga AA, Callaghan WM. Prevalence and patterns of marijuana use among pregnant and nonpregnant women of reproductive age. Am J Obstet Gynecol. 2015;213(2):201.e1-201.e10. doi:10.1016/j.ajog.2015.03.021 25772211 PMC7469257

[36] Gordon NP. Similarity of adult Kaiser Permanente members to the adult population in Kaiser Permanente’s Northern California service area: comparisons based on the 2017/2018 cycle of the California Health Interview Survey. Kaiser Permanente Division of Research. November 8, 2020. Accessed April 1, 2022. https://divisionofresearch.kaiserpermanente.org/projects/memberhealthsurvey/SiteCollectionDocuments/compare_kp_ncal_chis2017-18.pdf

[37] Goler NC, Armstrong MA, Osejo VM, Hung YY, Haimowitz M, Caughey AB. Early Start: a cost-beneficial perinatal substance abuse program. Obstet Gynecol. 2012;119(1):102-110. doi:10.1097/AOG.0b013e31823d427d 22183217

[38] Lieberman L, Taillac C, Goler N. Vision, research, innovation and influence: Early Start’s 15-year journey from pilot project to regional program. Perm J. 2005;9(1):62-64. doi:10.7812/TPP/04-134 21687486 PMC3108416

[39] Taillac C, Goler N, Armstrong MA, Haley K, Osejo V. Early Start: an integrated model of substance abuse intervention for pregnant women. Perm J. 2007;11(3):5-11. doi:10.7812/TPP/07-013 21461106 PMC3057720

[40] Young-Wolff KC, Sarovar V, Tucker LY, . Validity of self-reported cannabis use among pregnant females in Northern California. J Addict Med. 2020;14(4):287-292. doi:10.1097/ADM.0000000000000581 31688149 PMC7931632

[41] Xue X, Kim MY, Gaudet MM, . A comparison of the polytomous logistic regression and joint Cox proportional hazards models for evaluating multiple disease subtypes in prospective cohort studies. Cancer Epidemiol Biomarkers Prev. 2013;22(2):275-285. doi:10.1158/1055-9965.EPI-12-1050 23292084 PMC3565022

[42] Jarlenski M, Tarr JA, Holland CL, Farrell D, Chang JC. Pregnant women’s access to information about perinatal marijuana use: a qualitative study. Womens Health Issues. 2016;26(4):452-459. doi:10.1016/j.whi.2016.03.010 27131908 PMC4958505

[43] Jarlenski M, Koma JW, Zank J, Bodnar LM, Tarr JA, Chang JC. Media portrayal of prenatal and postpartum marijuana use in an era of scientific uncertainty. Drug Alcohol Depend. 2018;187:116-122. doi:10.1016/j.drugalcdep.2018.02.021 29655873 PMC5959784

[44] Dakkak H, Brown R, Twynstra J, Charbonneau K, Seabrook JA. The perception of pre- and post-natal marijuana exposure on health outcomes: a content analysis of Twitter messages. J Neonatal Perinatal Med. 2018;11(4):409-415. doi:10.3233/NPM-17133 29843262

[45] Centers for Disease Control and Prevention, Division of Reproductive Health, National Center for Chronic Disease Prevention and Health Promotion. Addressing opioid use disorder to improve maternal and infant health. Page last reviewed: March 30, 2022. Accessed April 6, 2022. https://www.cdc.gov/reproductivehealth/maternalinfanthealth/substance-abuse/opioid-use-disorder-pregnancy/addressing-opioid-use-maternal-infant-health.htm

[46] Centers for Disease Control and Prevention, Office on Smoking and Health, National Center for Chronic Disease Prevention and Health Promotion. Pregnant or planning to have a baby. Page last reviewed: February 11, 2021. Accessed April 6, 2022. https://www.cdc.gov/tobacco/campaign/tips/groups/pregnant-planning.html

[47] Mark K, Gryczynski J, Axenfeld E, Schwartz RP, Terplan M. Pregnant women’s current and intended cannabis use in relation to their views toward legalization and knowledge of potential harm. J Addict Med. 2017;11(3):211-216. doi:10.1097/ADM.0000000000000299 28252456

[48] Compton WM, Han B, Jones CM, Blanco C, Hughes A. Marijuana use and use disorders in adults in the USA, 2002-14: analysis of annual cross-sectional surveys. Lancet Psychiatry. 2016;3(10):954-964. doi:10.1016/S2215-0366(16)30208-5 27592339

[49] Holland CL, Rubio D, Rodriguez KL, . Obstetric health care providers’ counseling responses to pregnant patient disclosures of marijuana use. Obstet Gynecol. 2016;127(4):681-687. doi:10.1097/AOG.0000000000001343 26959210 PMC4805441

[50] Azofeifa A, Mattson ME, Schauer G, McAfee T, Grant A, Lyerla R. National estimates of marijuana use and related indicators—National Survey on Drug Use and Health, United States, 2002-2014. MMWR Surveill Summ. 2016;65(11):1-28. doi:10.15585/mmwr.ss6511a1 27584586

