# Supplementary material for: Trends in Cannabis Polysubstance Use During Early Pregnancy Among Patients in a Large Health Care System in Northern California
Source: JAMA Netw Open. 2022 Jun 6;5(6):e2215418. doi: 10.1001/jamanetworkopen.2022.15418 (PMC9171564; doi:10.1001/jamanetworkopen.2022.15418)
Supplement: Supplement. — eAppendix. Urine Toxicology Testing Description and Positive Cutoffs eTable 1. Prevalence of Cannabis Use and Polysubstance Use During Early Pregnancy in KPNC by Sociodemographics, 2009-2018 (N = 367,138) eTable 2. Adjusted Prevalence and Annual Relative Rate of Change of Cannabis, Alcohol, Nicotine, Pharmaceutical Opioid, and Stimulant Use During Early Pregnancy in KPNC, 2009-2018 (N = 367,138) eTable 3. Sensitivity Analysis of Adjusted Prevalence and Annual Relative Rate of Change of Cannabis Use During Early Pregnancy in KPNC for Each Year (2009-2018), by Polysubstance Use (N = 367,138) [file jamanetwopen-e2215418-s001.pdf]

Supplementary Online Content

Young-Wolff KC, Sarovar V, Tucker LY, et al. Trends in cannabis polysubstance use during early pregnancy among patients in a large health care system in Northern California. *JAMA Netw Open.* 2022;5(6):e2215418. doi:10.1001/jamanetworkopen.2022.15418

eAppendix. Urine Toxicology Testing Description and Positive Cutoffs
eTable 1. Prevalence of Cannabis Use and Polysubstance Use During Early Pregnancy in KPNC by Sociodemographics, 2009-2018 (N = 367,138)
eTable 2. Adjusted Prevalence and Annual Relative Rate of Change of Cannabis, Alcohol, Nicotine, Pharmaceutical Opioid, and Stimulant Use During Early Pregnancy in KPNC, 2009-2018 (N = 367,138)
eTable 3. Sensitivity Analysis of Adjusted Prevalence and Annual Relative Rate of Change of Cannabis Use During Early Pregnancy in KPNC for Each Year (2009-2018), by Polysubstance Use (N = 367,138)

This supplementary material has been provided by the authors to give readers additional information about their work.

© 2022 Young-Wolff KC et al. *JAMA Network Open.*

eAppendix. Urine Toxicology Testing Description and Positive Cutoffs

Cannabis: Cannabis screening tests were performed on a Beckman Coulter AU680 chemistry analyzer using the Emit II Plus Cannabinoid Assay from Siemens with a cutoff of 45ng/mL. Confirmatory testing for the presence of the cannabis metabolite, 11-nor-9-carboxy-delta 9- THC, was performed by liquid chromatography-tandem mass spectrometry for all positive immunoassay results. The confirmation test methodology was LC-MS/MS on a triple quadrupole system with a cutoff for positivity of 15ng/mL.

Alcohol: Alcohol screening tests were performed on a Beckman Coulter AU680 chemistry analyzer using the Emit II Plus Ethyl Alcohol Assay from Siemens with a cutoff of 10 mg/dL. Confirmatory testing for the presence of ethanol was performed by gas chromatography-mass spectrometry for all positive immunoassay results, also with a positive cutoff of 10 mg/dL.

Pharmaceutical Opioids: Opiate screening tests were performed on a Beckman Coulter AU680 chemistry analyzer using the Emit II Plus Opiate 300 Assay from Siemens with a cutoff of 300 ng/mL. Confirmatory testing for the presence of opiate species was performed by liquid chromatography-tandem mass spectrometry for all positive immunoassay results. Opiate species detected by the confirmation assay include morphine, codeine, hydrocodone, norhydrocodone, hydromorphone, oxycodone, noroxycodone, and oxymorphone. All species have a positive cutoff of 50 ng/mL.

Amphetamine/Methamphetamine: Amphetamines screening tests were performed on a Beckman Coulter AU680 chemistry analyzer using the DRI Amphetamines Assay with a cutoff of 500 ng/mL. Confirmatory testing for the presence of amphetamine species was performed by liquid

© 2022 Young-Wolff KC et al. *JAMA Network Open.*

chromatography- tandem mass spectrometry for all positive immunoassay results. Amphetamine species detected by the confirmation assay include amphetamine and methamphetamine. All species have a positive cutoff of 250 ng/mL.

Cocaine: Cocaine screening tests were performed on a Beckman Coulter AU680 chemistry analyzer using the Emit II Plus Cocaine Metabolite Assay which detects the cocaine metabolite benzoylecgonine with a positive cutoff of 150 ng/mL. Confirmatory testing for the presence of benzoylecgonine was performed by liquid chromatography- tandem mass spectrometry for all positive immunoassay results. The positive cutoff for the confirmation assay was 100 ng/mL.

© 2022 Young-Wolff KC et al. *JAMA Network Open.*

eTable 1. Prevalence of Cannabis Use and Polysubstance Use During Early Pregnancy in KPNC by Sociodemographics, 2009-2018 (N = 367,138)

Characteristics	Total	Any Cannabis Use		Number of Co-Occurring Substances			
	N (%) = 367,138 (100.0)	No n (%) = 344,893 (93.9)	Yes n (%) = 22,245 (6.1)	No Cannabis n (%) = 344,893 (93.9)	Cannabis Only n (%) = 15,488 (4.2)	Cannabis and 1 other Substance n (%) = 4,984 (1.4)	Cannabis and ≥2 Substances n (%) = 1,773 (0.5)
**Age, Years**							
11-17	4,170 (1.1)	3,427 (82.2)	743 (17.8)	3,427 (82.2)	510 (12.2)	168 (4.0)	65 (1.6)
18-24	54,639 (14.9)	46,079 (84.3)	8,560 (15.7)	46,079 (84.3)	6,133 (11.2)	1,729 (3.2)	698 (1.3)
25-34	227,226 (61.9)	216,740 (95.4)	10,486 (4.6)	216,740 (95.4)	7,257 (3.2)	2,427 (1.1)	802 (0.4)
≥35	81,103 (22.1)	78,647 (97.0)	2,456 (3.0)	78,647 (97.0)	1,588 (2.0)	660 (0.8)	208 (0.3)
**Race/Ethnicity**							
White	139,616 (38.0)	130,816 (93.7)	8,800 (6.3)	130,816 (93.7)	5,742 (4.1)	2,210 (1.6)	848 (0.6)
Black	24,378 (6.6)	19,158 (78.6)	5,220 (21.4)	19,158 (78.6)	3,921 (16.1)	1,001 (4.1)	298 (1.2)
Asian/Pacific Islander	95,168 (25.9)	93,932 (98.7)	1,236 (1.3)	93,932 (98.7)	749 (0.8)	337 (0.4)	150 (0.2)
Hispanic	94,740 (25.8)	88,937 (93.9)	5,803 (6.1)	88,937 (93.9)	4,250 (4.5)	1,165 (1.2)	388 (0.4)
Other/Unknown	13,236 (3.6)	12,050 (91.0)	1,186 (9.0)	12,050 (91.0)	826 (6.2)	271 (2.1)	89 (0.7)
**Median Neighborhood Household Income[a]**							
<51,563	91,589 (25.0)	82,769 (90.4)	8,820 (9.6)	82,769 (90.4)	6,409 (7.0)	1,819 (2.0)	592 (0.7)
51,563 -<70,455	91,574 (24.9)	85,749 (93.6)	5,825 (6.4)	85,749 (93.6)	4,036 (4.4)	1,319 (1.4)	470 (0.5)
70,455 - <92,625	91,569 (24.9)	87,133 (95.2)	4,436 (4.8)	87,133 (95.2)	2,976 (3.3)	1,055 (1.2)	405 (0.4)
≥ 92,625	91,602 (25.0)	88,491 (96.6)	3,111 (3.4)	88,491 (96.6)	2,040 (2.2)	773 (0.8)	298 (0.3)

Notes. [a] Data on neighborhood median household income was missing for 804 (0.2%) pregnancies.

© 2022 Young-Wolff KC et al. *JAMA Network Open.*

eTable 2. Adjusted Prevalence and Annual Relative Rate of Change of Cannabis, Alcohol, Nicotine, Pharmaceutical Opioid, and Stimulant Use During Early Pregnancy in KPNC, 2009-2018 (N = 367,138)

	Adjusted prevalence of any substance use during pregnancy, % (95% CI)[a]										Linear trend estimation	
Any use		2009	2010	2011	2012	2013	2014	2015	2016	2017	2018	Annual relative rate of change estimate (95% CI)	*P* value
Alcohol	11.73 (11.31-12.14)	10.72 (10.32-11.12)	10.55 (10.15-10.94)	10.08 (9.70-10.46)	9.57 (9.20-9.93)	8.98 (8.63-9.32)	8.91 (8.58-9.25)	8.00 (7.68-8.31)	8.84 (8.52-9.17)	10.86 (10.51-11.20)		
	Annual relative rate	0.91 (0.87-0.96)	0.98 (0.93-1.04)	0.96 (0.91-1.01)	0.95 (0.90-1.00)	0.94 (0.89-0.99)	0.99 (0.94-1.05)	0.90 (0.85-0.95)	1.11 (1.05-1.17)	1.23 (1.17-1.29)	0.98 (0.97-0.98)	<.001
Cannabis	4.14 (3.85-4.43)	4.55 (4.24-4.85)	5.06 (4.74-5.39)	5.16 (4.83-5.48)	5.38 (5.04-5.71)	5.98 (5.63-6.33)	6.43 (6.08-6.79)	6.81 (6.45-7.17)	7.76 (7.38-8.15)	8.73 (8.33-9.12)		
	Annual relative rate	1.10 (1.00-1.21)	1.11 (1.01-1.22)	1.02 (0.93-1.12)	1.04 (0.95-1.14)	1.11 (1.02-1.21)	1.08 (0.99-1.17)	1.06 (0.98-1.14)	1.14 (1.06-1.23)	1.12 (1.05-1.20)	1.08 (1.08 -1.09)	<.001
Nicotine	4.21 (3.95-4.48)	3.54 (3.29-3.78)	3.45 (3.21-3.69)	3.03 (2.80-3.25)	2.91 (2.68-3.13)	2.61 (2.40-2.81)	2.33 (2.14-2.52)	2.21 (2.03-2.39)	1.98 (1.81-2.15)	2.06 (1.89-2.22)		
	Annual relative rate	0.84 (0.76-0.92)	0.98 (0.88-1.08)	0.88 (0.79-0.97)	0.96 (0.86-1.07)	0.90 (0.80-1.00)	0.89 (0.80-1.00)	0.95 (0.84-1.06)	0.90 (0.80-1.01)	1.04 (0.92-1.17)	0.92 (0.91-0.93)	<.001
Pharmaceutical opioids[b]	0.52 (0.45-0.58)	0.43 (0.37-0.49)	0.47 (0.40-0.53)	0.48 (0.42-0.55)	0.53 (0.46-0.59)	0.48 (0.42-0.55)	0.48 (0.42-0.53)	0.38 (0.33-0.43)	0.26 (0.22-0.31)	0.22 (0.19-0.26)		
	Annual relative rate	0.84 (0.69-1.02)	1.08 (0.89-1.31)	1.04 (0.86-1.26)	1.09 (0.91-1.30)	0.92 (0.77-1.10)	0.98 (0.82-1.17)	0.80 (0.66-0.96)	0.70 (0.56-0.86)	0.85 (0.67-1.07)	0.94 (0.92-0.95)	<.001
Stimulants[c]	0.34 (0.28-0.40)	0.29 (0.24-0.35)	0.34 (0.28-0.40)	0.33 (0.27-0.38)	0.33 (0.28-0.39)	0.41 (0.35-0.48)	0.37 (0.31-0.43)	0.39 (0.33-0.45)	0.43 (0.37-0.50)	0.48 (0.42-0.55)		
	Annual relative rate	0.86 (0.67-1.11)	1.17 (0.91-1.50)	0.95 (0.74-1.22)	1.03 (0.80-1.32)	1.23 (0.98-1.55)	0.91 (0.73-1.13)	1.05 (0.84-1.31)	1.11 (0.90-1.36)	1.11 (0.91-1.35)	1.05 (1.03-1.06)	<.001

Notes. [a]Adjusted prevalence estimates and 95% confidence intervals (CIs) were estimated from Poisson regression models controlling for age group, race/ethnicity and median neighborhood household income (extracted from electronic health record). [b]Pharmaceutical opioid use is based on a urine toxicology test. [c]Stimulant use is based on a combination of self-report (cocaine and methamphetamine) and/or toxicology testing (cocaine and methamphetamine/amphetamine).

© 2022 Young-Wolff KC et al. *JAMA Network Open.*

eTable 3. Sensitivity Analysis of Adjusted Prevalence and Annual Relative Rate of Change of Cannabis Use During Early Pregnancy in KPNC for Each Year (2009-2018), by Polysubstance Use (N = 367,138)

	Adjusted Prevalence of Cannabis and Co-Occurring Substance Use During Pregnancy, % (95% CI)[a]										Linear Trend Estimation	
Substance Use	2009	2010	2011	2012	2013	2014	2015	2016	2017	2018	Annual Relative Rate of Change Estimate (95% CI)	*P* value
**Number of Co-Occurring Substances**												
Cannabis Only	2.43 (2.23-2.62)	2.89 (2.67-3.10)	3.25 (3.02-3.48)	3.37 (3.14-3.60)	3.66 (3.42-3.91)	4.27 (4.01-4.53)	4.73 (4.46-4.99)	5.13 (4.85-5.40)	5.89 (5.59-6.18)	6.32 (6.02-6.62)		
	Annual relative rate	1.19 (1.07-1.33)	1.13 (1.02-1.25)	1.04 (0.94-1.14)	1.09 (0.99-1.20)	1.17 (1.06-1.28)	1.11 (1.02-1.20)	1.08 (1.00-1.17)	1.15 (1.07-1.24)	1.07 (1.00-1.15)	1.11 (1.10-1.12)	<.001
Cannabis and 1 Other Substance[b,c]	1.17 (1.04-1.29)	1.15 (1.02-1.27)	1.27 (1.14-1.40)	1.26 (1.13-1.39)	1.27 (1.14-1.40)	1.29 (1.16-1.42)	1.30 (1.17-1.43)	1.28 (1.15-1.41)	1.45 (1.32-1.59)	1.92 (1.77-2.07)		
	Annual relative rate	0.98 (0.84-1.14)	1.11 (0.95-1.29)	1.00 (0.86-1.15)	1.01 (0.87-1.17)	1.01 (0.87-1.17)	1.01 (0.88-1.16)	0.98 (0.86-1.13)	1.14 (0.99-1.30)	1.32 (1.17-1.49)	1.04 (1.03-1.06)	<.001
Cannabis and ≥2 Other Substances[b,c]	0.55 (0.47-0.62)	0.52 (0.44-0.59)	0.54 (0.46-0.62)	0.52 (0.45-0.60)	0.44 (0.37-0.51)	0.43 (0.36-0.49)	0.41 (0.34-0.47)	0.40 (0.34-0.46)	0.42 (0.36-0.49)	0.49 (0.42-0.56)		
	Annual relative rate	0.94 (0.77-1.16)	1.05 (0.85-1.28)	0.97 (0.79-1.19)	0.85 (0.68-1.05)	0.96 (0.77-1.21)	0.96 (0.76-1.20)	0.98 (0.78-1.23)	1.06 (0.85-1.32)	1.15 (0.93-1.42)	0.97 (0.96-0.99)	0.001
**Type of Co-Occurring Substance Use**												
Cannabis Only	2.43 (2.23-2.62)	2.89 (2.67-3.10)	3.25 (3.02-3.48)	3.37 (3.14-3.60)	3.66 (3.42-3.91)	4.27 (4.01-4.53)	4.73 (4.46-4.99)	5.13 (4.85-5.40)	5.89 (5.59-6.18)	6.32 (6.02-6.62)		
	Annual relative rate	1.19 (1.07-1.33)	1.13 (1.02-1.25)	1.04 (0.94-1.14)	1.09 (0.99-1.20)	1.17 (1.06-1.28)	1.11 (1.02-1.20)	1.08 (1.00-1.17)	1.15 (1.07-1.24)	1.07 (1.00-1.15)	1.11 (1.10-1.12)	<.001
Cannabis and Alcohol	1.11 (0.99-1.23)	1.08 (0.96-1.21)	1.14 (1.01-1.27)	1.16 (1.03-1.28)	1.13 (1.01-1.26)	1.12 (0.99-1.24)	1.10 (0.98-1.22)	1.07 (0.96-1.19)	1.35 (1.22-1.48)	1.87 (1.73-2.02)		
	Annual relative rate	0.97 (0.83-1.14)	1.05 (0.90-1.23)	1.02 (0.87-1.19)	0.98 (0.84-1.14)	0.99 (0.84-1.15)	0.99 (0.85-1.15)	0.97 (0.84-1.13)	1.26 (1.09-1.45)	1.39 (1.23-1.57)	1.04 (1.03-1.06)	<.001
Cannabis and Nicotine	1.05 (0.93-1.16)	0.97 (0.86-1.09)	1.05 (0.93-1.17)	0.99 (0.88-1.11)	0.90 (0.79-1.01)	0.88 (0.77-0.98)	0.85 (0.74-0.95)	0.84 (0.74-0.94)	0.78 (0.68-0.88)	0.83 (0.73-0.92)		
	Annual relative rate	0.93 (0.79-1.09)	1.07 (0.91-1.26)	0.95 (0.81-1.11)	0.91 (0.77-1.07)	0.98 (0.82-1.16)	0.96 (0.81-1.15)	0.99 (0.84-1.18)	0.93 (0.78-1.10)	1.06 (0.89-1.26)	0.97 (0.96-0.98)	<.001
Cannabis and Illicit/Unauthorized Pharmaceutical Opioids[b]	0.01 (0.01-0.02)	0.03 (0.02-0.04)	0.02 (0.01-0.03)	0.03 (0.02-0.05)	0.05 (0.03-0.06)	0.05 (0.04-0.06)	0.06 (0.04-0.07)	0.05 (0.03-0.06)	0.04 (0.03-0.06)	0.04 (0.03-0.06)		

© 2022 Young-Wolff KC et al. *JAMA Network Open.*

	Annual relative rate	1.82 (0.96-3.45)	0.90 (0.52-1.58)	1.44 (0.85-2.44)	1.36 (0.88-2.11)	1.07 (0.72-1.59)	1.18 (0.82-1.71)	0.80 (0.56-1.16)	0.94 (0.64-1.38)	1.00 (0.68-1.47)	1.09 (1.06-1.13)	<.001
Cannabis and Illicit/Unauthorized Stimulants[c]	0.16 (0.12-0.20)	0.15 (0.11-0.18)	0.20 (0.16-0.24)	0.17 (0.13-0.20)	0.15 (0.11-0.18)	0.17 (0.13-0.21)	0.18 (0.14-0.22)	0.17 (0.14-0.21)	0.18 (0.14-0.22)	0.21 (0.17-0.25)		
	Annual relative rate	0.90 (0.65-1.26)	1.37 (0.99-1.88)	0.84 (0.62-1.14)	0.89 (0.64-1.23)	1.17 (0.84-1.62)	1.03 (0.75-1.39)	0.98 (0.72-1.33)	1.05 (0.78-1.42)	1.14 (0.86-1.51)	1.02 (1.00-1.05)	0.070

Notes. [a]Adjusted prevalence estimates and 95% CIs were estimated from Poisson regression models controlling for age group, race/ethnicity, and median neighborhood household income (extracted from the electronic health record). [b]Illicit/unauthorized pharmaceutical opioid use was defined as having a positive screen for prescription opioid use by urine toxicology and not having a prescription for an opioid filled during the 90 days prior to the screening date. [c]Illicit/unauthorized stimulant use was defined as (1) cocaine and/or methamphetamine use by self-report and/or urine toxicology testing, and/or (2) a positive toxicology test for amphetamines and no prescription for an amphetamine filled during the 90 days prior to the screening date.

© 2022 Young-Wolff KC et al. *JAMA Network Open.*
